# A RIPK3-independent role of MLKL in suppressing parthanatos promotes immune evasion in hepatocellular carcinoma

**DOI:** 10.1038/s41421-022-00504-0

**Published:** 2023-01-17

**Authors:** Xifei Jiang, Wenjia Deng, Siyao Tao, Zheng Tang, Yuehong Chen, Mengxin Tian, Ting Wang, Chenyang Tao, Yize Li, Yuan Fang, Congying Pu, Jun Gao, Xiaomin Wang, Weifeng Qu, Xiameng Gai, Zhenbin Ding, Yixian Fu, Ying Zheng, Siyuwei Cao, Jian Zhou, Min Huang, Weiren Liu, Jun Xu, Jia Fan, Yinghong Shi

**Affiliations:** 1 Department of Liver Surgery and Transplantation, Liver Cancer Institute, Zhongshan Hospital, Fudan University & Research Unit of Liver Cancer Recurrence and Metastasis, Chinese Academy of Medical Sciences, Shanghai, China; 2 Key Laboratory of Carcinogenesis and Cancer Invasion of Ministry of Education, Shanghai, China; 3 State Key Laboratory of Drug Research, Shanghai Institute of Materia Medica, Chinese Academy of Sciences, Shanghai, China; 4 University of Chinese Academy of Sciences, Beijing, China; 5 Department of General Surgery, Zhongshan Hospital, Fudan University, Shanghai, China; 6 Institute of Biomedical Sciences, Fudan University, Shanghai, China; 7 Shanghai Key Laboratory of Organ Transplantation, Shanghai, China; 8 State Key Laboratory of Genetic Engineering and Collaborative Innovation Center for Genetics and Development, School of Life Sciences, Fudan University, Shanghai, China

**Keywords:** Cancer, Cell death

## Abstract

Mixed lineage kinase domain-like (MLKL) is widely accepted as an executioner of necroptosis, in which MLKL mediates necroptotic signaling and triggers cell death in a receptor-interacting protein kinase 3 (RIPK3)-dependent manner. Recently, it is increasingly noted that RIPK3 is intrinsically silenced in hepatocytes, raising a question about the role of MLKL in hepatocellular carcinoma (HCC). This study reports a previously unrecognized role of MLKL in regulating parthanatos, a programmed cell death distinct from necroptosis. In HCC cells with intrinsic RIPK3 deficiency, knockout of MLKL impedes the orthotopic tumor growth, activates the anti-tumor immune response and enhances the therapeutic effect of immune checkpoint blockade in syngeneic HCC tumor models. Mechanistically, MLKL is required for maintaining the endoplasmic reticulum (ER)-mitochondrial Mg^2+^ dynamics in HCC cells. MLKL deficiency restricts ER Mg^2+^ release and mitochondrial Mg^2+^ uptake, leading to ER dysfunction and mitochondrial oxidative stress, which together confer increased susceptibility to metabolic stress-induced parthanatos. Importantly, pharmacological inhibition of poly(ADP-ribose) polymerase to block parthanatos restores the tumor growth and immune evasion in MLKL-knockout HCC tumors. Together, our data demonstrate a new RIPK3-independent role of MLKL in regulating parthanatos and highlight the role of MLKL in facilitating immune evasion in HCC.

## Introduction

Regulated cell death (RCD) is a fundamental biological process that underlies many physiological and pathological traits, including organismal development, tissue homeostasis, and immune responses. Apart from the well-understood apoptosis, a growing number of novel non-apoptotic forms of RCD, such as necroptosis, pyroptosis, ferroptosis and parthanatos, have been identified lately. Different forms of RCD are characterized by distinct morphological, biochemical and molecular features, yet extensive cross-talks are also identified between each other^1^. Cancer cells undergoing RCD may produce immunostimulatory effects via releasing numerous immunogenic contents that stimulate the dendritic cells to prime the adaptive immune cells for anti-cancer activity. As malignant cells are well-known for developing different approaches to escape immune surveillance, harnessing the immunogenic potential of cancer cells to overcome their immune-evasion phenotype has emerged as a new strategy for effective cancer therapy.

RCD of hepatocytes is a common trigger for liver diseases arising from different etiologies. Over 80% of hepatocellular carcinoma (HCC), the most prevalent type of primary liver cancer, occurs in the context of a fibrotic or cirrhotic liver characterized by substantial hepatocellular cell death and inflammation. Different types of cell death have been linked to the progression of liver disease and the development of HCC, among which apoptosis and necroptosis are the most characterized. While apoptosis has been associated with a key mechanism to prevent malignancy, necroptosis is largely considered as an immunogenic form of cell death that might be involved in the modulation of both tumor cell proliferation and immune cell activation. Whether other types of RCD are involved in HCC progression remains unknown^2^.

Necroptosis is one of the best-understood forms of RCD. Necroptosis typically occurs in a specific context where apoptotic signaling is inhibited, during which death receptor triggers the formation of RIPK1/RIPK3 complex and subsequent phosphorylation of pseudokinase mixed lineage kinase domain-like protein (MLKL). RIPK3-dependent phosphorylation of MLKL results in a conformational switch and translocation of MLKL from cytosol to diverse cellular membranes, including the plasma membrane where it causes loss of membrane integrity and eventually necrotic death^1,3–5^. Of note, there is a vigorous controversy over the roles of RIPK3 in hepatocytes and the associated liver diseases^6,7^. Accumulative evidence has suggested that hepatocellular necroptosis plays an important role in liver diseases^8^. Blockage of RIPK3-mediated necroptosis ameliorates liver injury and fibrosis^8–11^. On the other hand, it is increasingly noted that RIPK3 is epigenetically silenced in primary hepatocytes in multiple liver disease models and prevents MLKL-mediated cell necroptosis^5,12,13^.

This study was intrigued by the discrepancy in the role of RIPK3 in hepatic diseases. We discovered that HCC cells are characterized by intrinsic RIPK3 deficiency, which allowed us to uncover a RIPK3-independent function of MLKL in HCC. We herein provide the first evidence showing that MLKL regulates parthanatos and the associated immune response in HCC.

## Results

### MLKL is required for the orthotopic tumor growth of RIPK3-deficient HCC

**Fig. 1:**
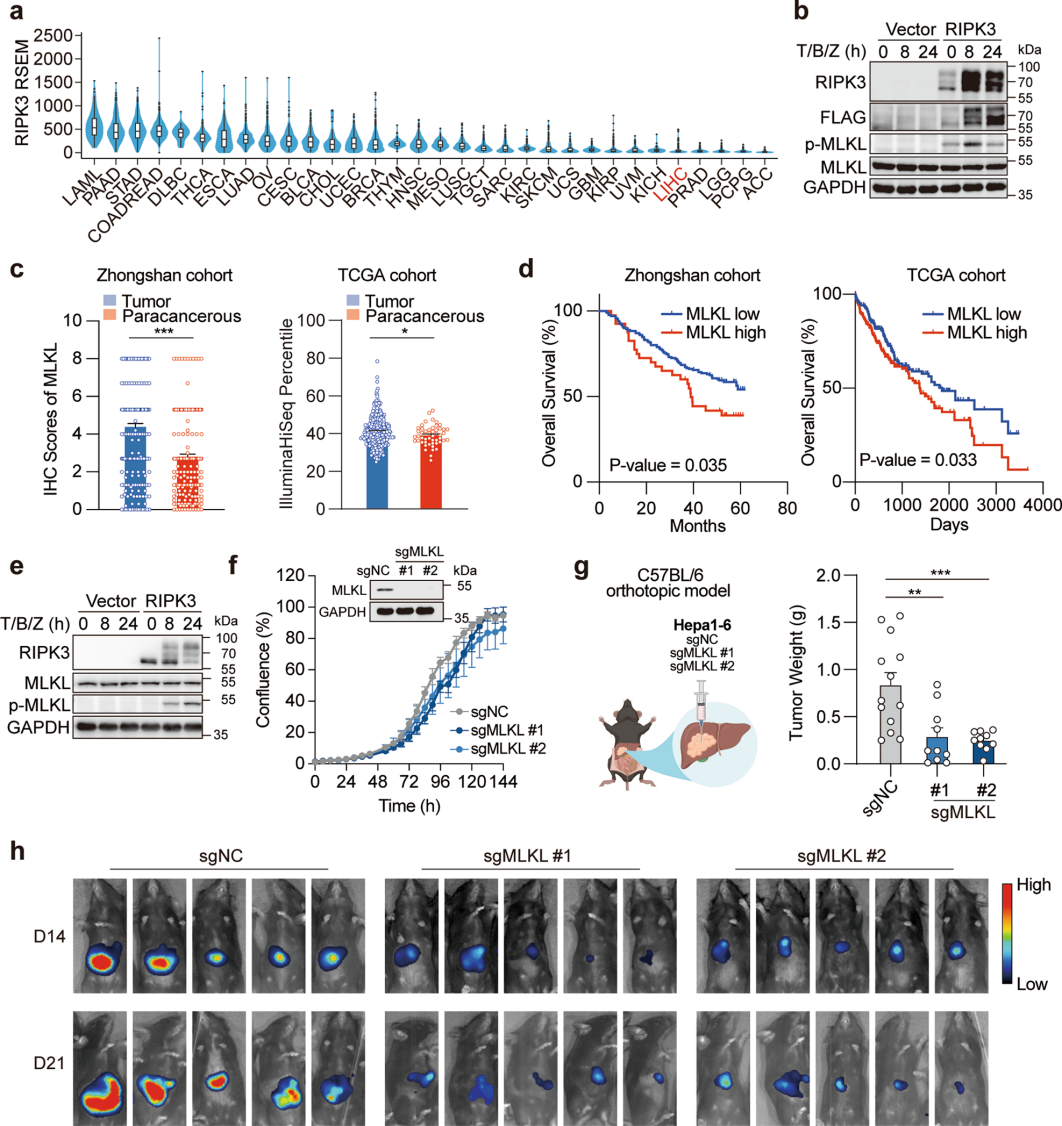
MLKL deficiency impairs the orthotopic growth of HCC tumors. **a** RIPK3 expression in patient samples across different types of cancer. Data were extracted from TCGA database. **b** Reconstitution of RIPK3 restores the necroptotic signaling in human HCC cells. HepG2 cells were transfected with the empty vector or pcDNA3.1-hRIPK3-Flag for 24 h and necroptotic signaling was stimulated by 50 μM Z-VAD-FMK followed by 50 ng/mL TNFα plus 20 μM Birinapant (T/B/Z). **c** MLKL expression in cancer tissues and adjacent normal tissues in two independent cohorts of HCC patients. **d** Survival analysis of MLKL high- and low-expression patients. The same set of data as in **c** were used for the analysis. **e** Reconstitution of RIPK3 restores the necroptotic signaling in murine HCC cells. Hepa 1–6 cells were treated and analyzed as described in **b**. **f** Growth curves of MLKL-KO and control murine HCC cells in vitro. Hepa 1–6-luc cells were infected with lentivirus-delivered sgMLKL or negative control (sgNC). KO efficiency was assessed by immunoblotting and cell growth was monitored by the live-cell analysis system IncuCyte (*n* = 3). **g**, **h** Orthotopic tumor growth of MLKL-KO and control HCC cells in a syngeneic model. Cells as described in **f** were orthotopically implanted in the liver of C57BL/6 mice (*n* = 13 for sgNC; *n* = 10 for sgMLKL #1 and sgMLKL #2). Tumor growth was monitored by whole-animal imaging. Endpoint tumor weight was shown in **g**. Representative luminescent images were shown in **h**. Data are represented as means ± SEM. Two-tailed Student’s *t*-test was used for statistical analysis. ns, not significant; *P < 0.05, **P < 0.01, ***P < 0.001.

Contradictory evidence has been noted for the roles of RIPK3 in hepatocytes. To confirm the RIPK3 status in HCC, we first examined the expression level of RIPK3 in a panel of HCC cell lines. HT29, a colon cancer cell line that is widely used for RIPK3-necroptosis study, was used as a positive control. The results showed that HCC cells barely expressed RIPK3 (Supplementary Fig. S1a). Consistent with this result, both cell-based data from Cancer Cell Line Encyclopedia (Supplementary Fig. S1b) and HCC patient sample data from The Cancer Genome Atlas (TCGA) (Fig. 1a) indicated that RIPK3 expression was at a very low level in HCC samples.

To investigate whether the undetectable RIPK3 in HCC may result in the deficiency in the necroptotic signaling in these cells, human HCC HepG2 cells were exposed to a combination treatment of TNF-α, birinapant and Z-VAD-FMK, a classic necroptosis-inducing condition. Meanwhile, human RIPK3 was introduced into these cells for comparison. In fact, HepG2 cells barely responded to the induction of necroptosis, while the reconstitution of RIPK3 evidently restored the necroptotic signaling, as indicated by the activation of MLKL, the executioner of necroptosis (Fig. 1b). These results echo the previous findings and confirm the intrinsic deficiency of RIPK3 and RIPK3-dependent necroptosis in HCC.

We were intrigued to ask whether MLKL plays a role in RIPK3-deficient HCC. We first explored the association between MLKL expression and patient prognosis in HCC using two independent HCC patient cohorts, a Chinese cohort composed of 211 HCC patients (Zhongshan cohort) and a TCGA cohort including both Caucasian and Asian patients. In Zhongshan cohort, MLKL expression in HCC specimens and corresponding adjacent normal tissues was analyzed by immunohistochemistry (Supplementary Fig. S1c) and patient follow-up data were obtained after curative hepatectomy. MLKL expression in cancer tissues was significantly higher than that in adjacent normal tissues (Fig. 1c). MLKL expression level in tumor tissues was significantly associated with the patient survival (Fig. 1d). Similar results were obtained by analyzing transcriptional levels of MLKL in TCGA cohort (Fig. 1c, d).

This result encouraged us to explore the RIPK3-independent role of MLKL in HCC. To this end, a widely used murine HCC cell line, Hepa 1–6, was particularly chosen to allow assessing the associated immune responses in vivo in the following study. Consistent with our data in human HCC cells, Hepa 1–6 cells showed defective response to necroptosis induction, whereas the introduction of human RIPK3 into these cells restored the necroptotic signaling (Fig. 1e). Then, MLKL-knockout (KO) luciferase-expressing Hepa 1–6 (Hepa 1–6-luc) cells were generated through CRISPR/Cas9-mediated gene editing using two independent single guide RNAs (sgMLKL #1 and sgMLKL #2), and the empty vector was used as a negative control (sgNC) (Fig. 1f). In these cells, MLKL depletion did not affect the cell growth in vitro (Fig. 1f). Of note, orthotopic inoculation of these cells in the liver of C57BL/6 mice resulted in a remarkable difference in tumor growth, as manifested by both luciferase intensity and the tumor weight at the endpoint of the study. Tumor growth was severely suppressed in MLKL-KO group (Fig. 1g, h; Supplementary Fig. S1d). Interestingly, when these cells were implanted in the flank of C57BL/6 mice, the difference in the tumor growth was abolished (Supplementary Fig. S1e, f). These results show that RIPK3 and its orchestrated necroptotic signaling are frequently deficient in HCC cells, and MLKL is required for the orthotopic growth of RIPK3-deficient tumors.

### MLKL deficiency activates antitumor immune response and sensitizes HCC tumors to immune checkpoint blockade

**Fig. 2:**
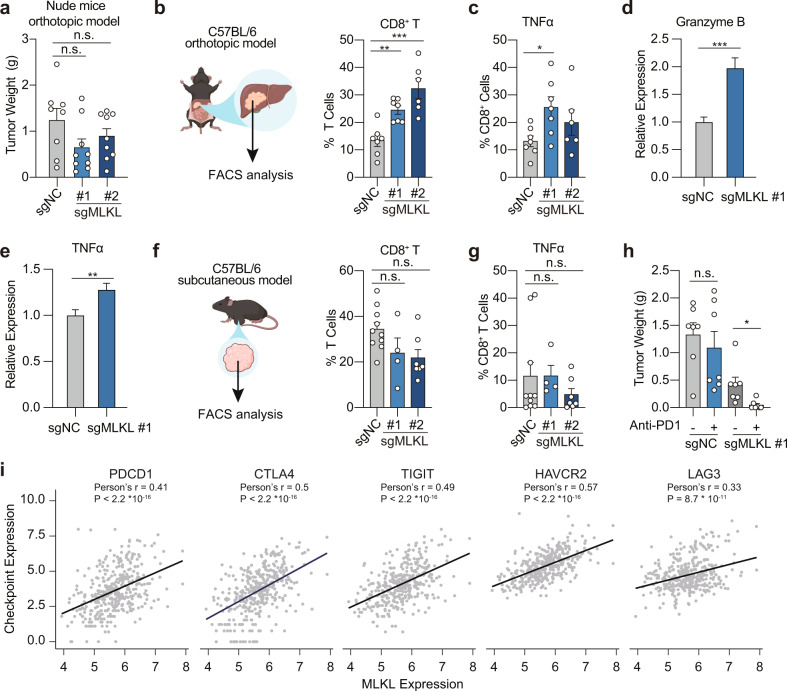
MLKL deficiency activates anti-tumor immunity in HCC tumors. **a** Endpoint tumor weight of MLKL-KO and control murine HCC cells in nude mice. MLKL-KO Hepa 1–6-luc (sgMLKL) or control (sgNC) cells were orthotopically implanted in the liver of nude mice (*n* = 8 for sgNC; *n* = 9 for sgMLKL #1 and sgMLKL #2). **b**, **c** Percentage of CD8^+^ T cells and TNFα^+^ CD8^+^ T cells in orthotopic tumors. MLKL-KO and control cells as described in **a** were orthotopically implanted in the liver of C57BL/6 mice and tumor-infiltrating immune cells were analyzed. **d**, **e** Relative expression of Granzyme B and TNFα in tumor-infiltrating immune cells as described in **b**. **f**, **g** Percentage of CD8^+^ T cells and TNFα^+^ CD8^+^ T cells in subcutaneous tumors. MLKL-KO and control cells as described in **a** were subcutaneously implanted in the right flank of C57BL/6 mice and tumor-infiltrating immune cells were analyzed. **h** Endpoint tumor weight upon treatment of anti-PD-1. Mice carrying MLKL-KO or control tumors as described in **b** were treated with anti-PD-1 antibody or isotype control antibodies every 3 days for 21 days (*n* = 7). **i** Correlation between checkpoint expression and MLKL expression in HCC patients. Data were from TCGA public database. Data are represented as means ± SEM. Two-tailed Student’s *t*-test was used for statistical analysis. ns, not significant; *P < 0.05, **P < 0.01, ***P < 0.001.

Our results above reveal an impact of MLKL on HCC tumor growth that requires its interaction with the local microenvironment in the liver. To explore how the tumor microenvironment was involved, we first asked whether the anti-tumor immunity was involved. MLKL-KO and control cells were orthotopically implanted in the immune-deficient nude mice. In great contrast to those implanted in the immune-competent mice, MLKL deficiency in HCC cells barely affected the tumor growth (Fig. 2a), suggesting the engagement of T cell response. We thus analyzed the tumor-infiltrating lymphocytes in Hepa 1–6 tumors. As expected, MLKL-KO tumors exhibited an increased proportion of cytotoxic CD8^+^ T cells, the major effector cells of the adaptive immune system against tumors, compared with the counterpart control group (Fig. 2b, c; Supplementary Fig. S2a). The increased CD8^+^ T cell infiltration was associated with enhanced cytotoxicity, as indicated by the expression of granzyme B and TNF-α (Fig. 2d, e). Meanwhile, cell surface markers indicating the maturity of innate immune cells, including myeloid dendritic cells and macrophages, were upregulated (Supplementary Fig. S2b–d), suggesting that antigen-presenting cells are probably involved to prime the adaptive immune cells. In contrast, CD4^+^ T cell infiltration was barely affected by the MLKL deficiency (Supplementary Fig. S2e). Importantly, these alterations were abolished when tumors were subcutaneously implanted in the immune-competent mice (Fig. 2f, g), which echoed the tumor growth phenotypes and suggested that the enhanced anti-tumor immune response in the liver may account for the impeded tumor growth upon MLKL depletion.

Immune checkpoint inhibitors, especially programmed death-1 (PD-1) inhibitors, have produced encouraging results in HCC patients^14^, yet only in a proportion of patients, highlighting the urgent need to identify biomarkers for patient selection. We next treated MLKL-proficient and -deficient tumor-bearing mice with anti-PD-1 antibody. Anti-PD-1 therapy alone was barely responded in Hepa 1–6 tumors, while the treatment in MLKL-deficient tumors resulted in apparent tumor regression (Fig. 2h). We further explored the relevance of MLKL with immune-responsive status in HCC by exploring TCGA public database. Consistent with our findings, the expression of MLKL in HCC was highly correlated with expression of multiple immune checkpoints including PD-1, CTLA-4, TIGIT, TIM3 and LAG3, suggesting the associated immune tolerance in MLKL-high expression tumors (Fig. 2i). These results together show that MLKL deficiency in HCC activates the antitumor immunity specifically in the hepatic tumor microenvironment, which accounts for the retarded tumor growth.

### MLKL protects HCC cells from undergoing metabolic stress-induced parthanatos

**Fig. 3:**
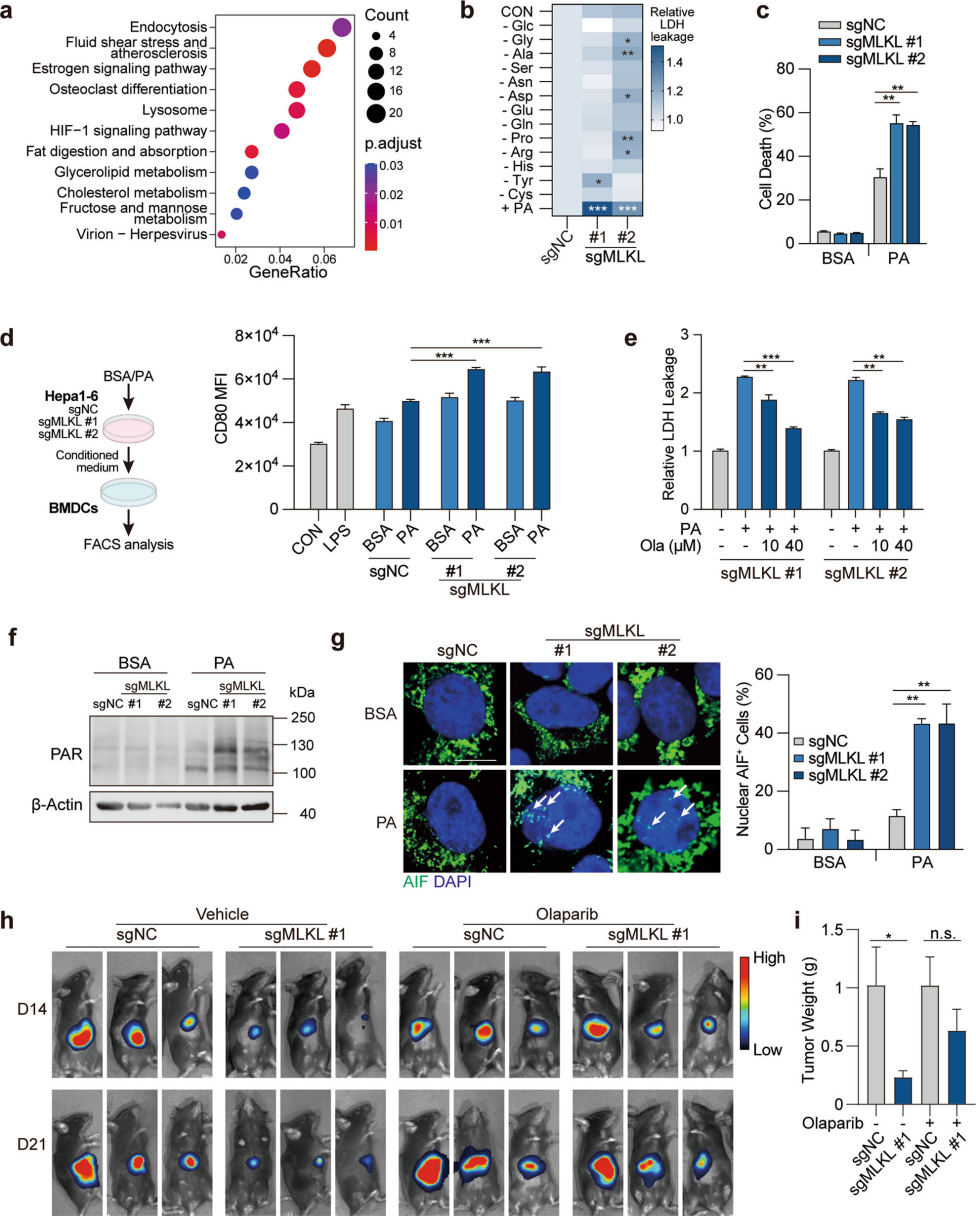
MLKL deficiency enhances metabolic stress-induced parthanatos. **a** KEGG pathway enrichment analysis of transcriptome data. MLKL-KO Hepa 1–6-luc (sgMLKL) or control (sgNC) cells were orthotopically implanted in the liver of C57BL/6 mice. Tumors collected at the endpoint of the study were subjected to RNA-seq analysis. **b** Cell death upon metabolic stress in HCC cells. MLKL-KO and control Hepa 1–6 cells were challenged with indicated nutrient deprivation or lipid stress (0.2 mM PA) for 24 h and cell death was measured by LDH leakage. **c** Cell death induced by PA. MLKL-KO and control Hepa 1–6 cells were treated with BSA or 0.2 mM PA for 24 h. Cell death was measured by SYTOX Green assay. **d** Dendritic cell activation. MLKL-KO Hepa 1–6 and control cells were treated with or without PA (0.2 mM, 24 h) and supernatant was collected as conditioned medium. BMDCs isolated from C57BL/6 mice were treated with conditioned medium for 24 h. Left, schematic diagram of the assay. Right, CD80 expression was analyzed using flow cytometry analysis. **e** PARP inhibition reverses cell death in MLKL-deficient cells. MLKL-KO and control Hepa 1–6 cells were treated with 0.2 mM PA and/or Olaparib at indicated concentrations for 24 h. **f** Immunoblotting analysis of PAR polymer accumulation. MLKL-KO and control Hepa 1–6 cells were treated with BSA or 0.2 mM PA for 20 h and then analyzed by immunoblotting. **g** AIF staining. Cells were treated as in **f** and AIF was stained using immunofluorescence. Left, representative images, scale bar, 10 μm. Arrows indicate AIF staining in the nucleus. Right, quantification of nuclear AIF positive cells. **h** Orthotopic tumor growth of MLKL-KO and control murine HCC cells. Cells were orthotopically implanted in the liver of C57BL/6 mice (*n* = 6). Mice were treated with or without Olaparib (50 mg/kg) daily. Tumor growth was monitored by whole-animal imaging. Shown were representative luminescent images. **i** Endpoint tumor weight as in **h**. Data are represented as means ± SEM. Two-tailed Student’s *t*-test was used for statistical analysis. ns, not significant; *P < 0.05, **P < 0.01, ***P < 0.001.

We further asked how MLKL deficiency in cancer cells provokes anti-tumor immunity specifically in the hepatic tumor microenvironment. To this end, orthotopic MLKL-KO Hepa 1–6 and control tumors collected from immune-competent C57BL/6 mice were subjected to RNA-seq analysis and subsequent KEGG pathway enrichment analysis (Fig. 3a). Pathway analysis of altered genes between MLKL-KO and the control group identified a set of pathways upregulated in MLKL-KO tumors, among which most are metabolism-related pathways, particularly lipid metabolism (Fig. 3a), suggesting that metabolic stress in the orthotopic tumors might be involved in the observed phenotypes.

Tumors in the liver microenvironment are continuously exposed to metabolic stress. In addition to the fierce competition for nutrients like glucose and non-essential amino acids, as other cancer types, the liver microenvironment is characterized by the metabolic stress caused by the excessive accumulation of saturated fatty acids^15–17^. To simulate metabolic stress in the liver tumor microenvironment, MLKL-KO and control cells were challenged in the culture deprived of glucose, different types of non-essential amino acids, or supplemented with palmitic acid (16:0, PA), the most abundant saturated fatty acids existing in the human body and closely linked to hepatic diseases^18^. Interestingly, among these conditions, only PA treatment specifically induced the cell death in MLKL-deficient cells, as indicated by the increased LDH leakage from the cells (Fig. 3b). PA treatment induced cell death in MLKL-deficient cells in a time- and dose-dependent manner (Supplementary Fig. S3a). Consistent with this result, SYTOX green nucleic acid staining detected more severely damaged integrity of the plasma membranes upon PA treatment in MLKL-deficient cells compared with the control cells (Fig. 3c). To further address whether PA-induced cell death contributed to the activation of immune response, the conditioned medium from PA-treated HCC cells were collected to treat bone marrow-derived dendritic cells (BMDCs). The medium from MLKL-deficient HCC cells were more effective in upregulating the maturity markers of BMDCs compared with that from the MLKL-proficient counterpart (Fig. 3d; Supplementary Fig. S3b). In contrast, MLKL-deficient and -proficient HCC cells showed a similar effect on CD8^+^ T cell activation (Supplementary Fig. S3c), suggesting that the elevated CD8^+^ T cells observed in MLKL-KO orthotopic tumors may be a consequence of antigen-presenting cell activation. These results strongly suggest the involvement of immunogenic cell death.

Apoptosis, necroptosis, pyroptosis and ferroptosis are well-known RCD forms that share the same phenomenon of leakage of immunogenic cytosolic components. We hence applied specific inhibitors (Z-VAD-FMK, necrostatin, ferrostatin) towards different types of cell death to examine whether they were able to rescue PA-induced cell death in MLKL-deficient HCC cells, yet none of the inhibitors showed the apparent impact (Supplementary Fig. S3d). As it lacks well-validated inhibitors for pyroptosis, we measured GSDMD and GSDME cleavage, two well-understood markers of pyroptosis, in PA-treated Hepa 1–6 cells pair, and the result largely excluded the involvement of pyroptosis (data not shown).

Recently, parthanatos is reported as a unique type of RCD associated with the increased oxidative stress, synthesis and accumulation of ploy(ADP-ribose) (PAR) polymer and nuclear translocation of Apoptosis inducing factor (AIF)^19^. To investigate whether parthanatos was involved, PARP (poly(ADP-ribose) polymerase) inhibitor olaparib was used to block parthanatos, which strikingly rescued the enhanced cell death in MLKL-deficient cells (Fig. 3e). Consistently, mitochondrial oxidative stress (Supplementary Fig. S3e), accumulation of PAR polymer (Fig. 3f; Supplementary Fig. S3f, g), and nuclear translocation of AIF (Fig. 3g) were all elevated in PA-challenged MLKL-deficient cells, together supporting the enhanced parthanatos in MLKL-deficient cells upon metabolic stress. In accordance with this result, MLKL-KO orthotopic tumors showed the increased level of parthanatos as well (Supplementary Fig. S3h, i). Moreover, MLKL-KO and control HCC cells were exposed to *N*-methyl-*N*′-nitro-*N*′-nitrosoguanidine (MNNG), another well-validated parthanatos inducer. Similarly, MNNG induced more severe cell death in MLKL-KO HCC cells (Supplementary Fig. S3j). Further, we tested whether this finding could be recapitulated in human HCC cells. In human HCC SK-HEP1 cells, siRNA-mediated MLKL-depletion increased the nuclear translocation of AIF (Supplementary Fig. S3k).

To further confirm that the enhanced parthanatos in MLKL-KO cells was independent of its well-defined role in necroptosis, MLKL mutant (T357A/S358A, A/A) that could not be activated by RIPK3, and the phosphomimic mutant (T357E/S358D, E/D) were overexpressed in MLKL-KO HCC cells. Both mutants rescued PA-induced PAR polymer accumulation in MLKL-KO cells to a similar extent (Supplementary Fig. S3l), suggesting that MLKL phosphorylation was not involved in this process. This result further strengthened a RIPK3-independent new role of MLKL in regulating parthanatos. Of great interest, we also noticed that the reconstitution of RIPK3 in Hepa 1–6 cells also reduced the parthanatos level (Supplementary Fig. S3m), suggesting that RIPK3 deficiency is likely required for the occurrence of parthanatos in this context.

To further demonstrate whether the enhanced parthanatos accounts for the impeded tumor growth, mice carrying orthotopic MLKL-KO and control Hepa 1–6 tumors were treated with PARP inhibitors in vivo. The results showed that PARP inhibition substantially restored the tumor growth (Fig. 3h, i) of MLKL-KO tumors, which was associated with the reversed PAR polymer accumulation (Supplementary Fig. S3n) and CD8^+^ T cell infiltration (Supplementary Fig. S3o). These results indicate that MLKL-deficient HCC cells undergo parthanatos to enhance anticancer immune surveillance.

### MLKL deficiency results in endoplasmic reticulum dysfunction

**Fig. 4:**
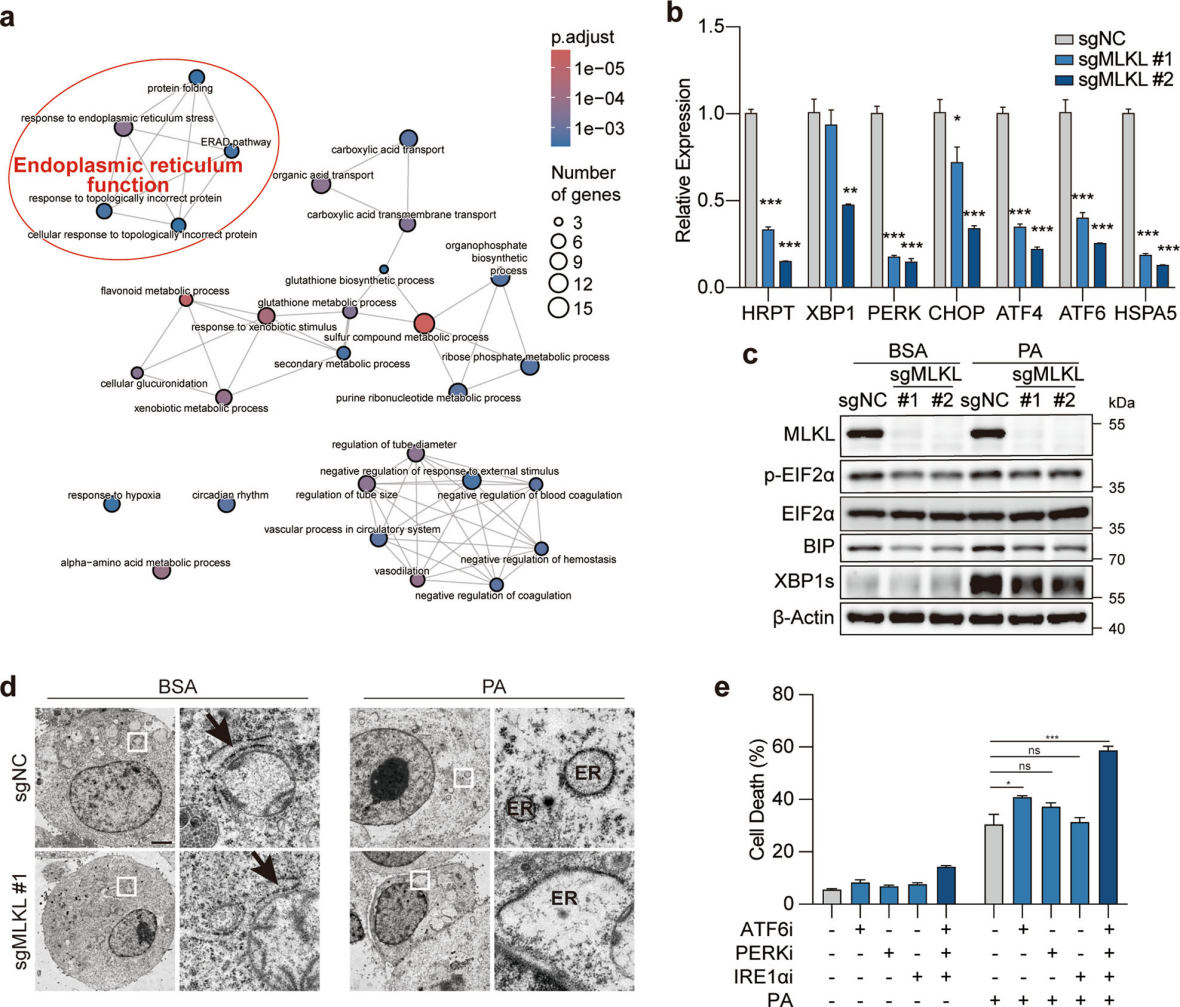
MLKL deficiency leads to ER dysfunction in HCC cells. **a** GO pathway enrichment analysis. MLKL-KO Hepa 1–6-luc (sgMLKL) or control (sgNC) cells were treated with BSA or 0.2 mM PA for 12 h and cells were collected for RNA-seq analysis. **b**, **c** mRNA and protein level validation of genes involved in ER stress. MLKL-KO Hepa 1–6 and control cells were subjected to qPCR analysis (**b**). Cells treated with BSA or PA (0.2 mM, 12 h) were subjected to immunoblotting analysis (**c**). **d** Transmission electron microscopy of ER. MLKL-KO Hepa 1–6-luc and control cells were treated with BSA or 0.2 mM PA for 4 h. Shown are representative images, scale bar, 200 nm. Arrows indicate ER. **e** Cell death analysis. Hepa 1–6 cells were pretreated with indicated inhibitors for 2 h and then challenged with 0.2 mM PA for 24 h and cell death was analyzed by SYTOX Green assay. Ceapin-A7 as ATF6 inhibitor (ATF6i, 20 μM), GSK2606414 as PERK inhibitor (PERKi, 20 μM) and 4μ8C as IRE1α inhibitor (IRE1αi, 20 μM). Data are represented as means ± SEM. Two-tailed Student’s *t*-test was used for statistical analysis. ns, not significant; *P < 0.05, **P < 0.01, ***P < 0.001.

We next asked how MLKL is functionally linked to parthanatos. To this end, RNA-seq analysis was performed in MLKL-proficient or -deficient Hepa1–6 cells with or without PA treatment. Through differential gene expression analysis, 161 genes were found upregulated upon PA challenge yet decreased in MLKL-deficient cells compared with MLKL-proficient counterparts. Gene Ontology (GO) pathway enrichment analysis of these genes revealed the enrichment of genes/pathways regulating endoplasmic reticulum (ER) function being down-regulated in MLKL-KO cells (Fig. 4a; Supplementary Fig. S4a). These results were confirmed using qPCR analysis (Fig. 4b).

As one of the largest organelles in eukaryotic cells, ER plays a major role in the synthesis, folding and structural maturation of more than a third of all proteins in the cell. PA is known for inducing ER stress that could activate the unfolded protein response (UPR) to re-establish ER homeostasis^20^. We hence examined the activation of UPR in these cells. As expected, PA treatment led to the activation of sequential molecular events in UPR, including expression of binding immunoglobulin protein (BIP), phosphorylation of EIF2α and cleavage of X-box binding protein 1 (XBP1), indicating the activation of UPR. However, UPR signaling activation was impaired when MLKL was depleted in these cells (Fig. 4c). To examine whether the function of ER was impaired upon MLKL depletion, the structure of ER was visualized using transmission electron micrographic analysis and fluorescent analysis in parallel. We observed that the network of the ER was broken into puncta-like structure upon PA treatment in the control cells, consistent with reported ER morphology under stress^21^. Of note, PA challenge triggered the formation of large swelling structure of ER in MLKL-deficient cells (Fig. 4d; Supplementary Fig. S4b, c).

We further examined whether the disruption of ER function could phenocopy PA-induced cell death specifically in MLKL-KO cells. To this end, PERK, IRE1α and ATF6, three ER-transmembrane sensors mediating the activation of UPR pathway, were individually inhibited by widely used inhibitors^22^. The results showed that neither individual nor combined inhibition of these proteins in Hepa 1–6 cells induced apparent cell death, yet PA treatment significantly enhanced cell death in cells exposed to combined inhibition (Fig. 4e). These results together suggest that MLKL-KO cells exhibit ER dysfunction and are more susceptible to metabolic stress-induced parthanatos.

### MLKL is critical for maintaining ER-mitochondrial Mg^2+^ dynamics in HCC cells

**Fig. 5:**
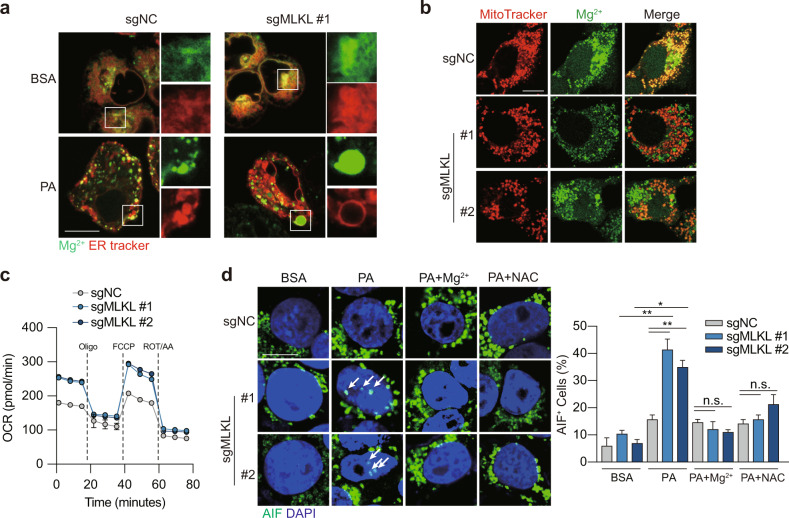
MLKL deficiency disrupts Mg^2+^ homeostasis in the ER. **a** Mg^2+^ signaling in ER. MLKL-KO Hepa 1–6 and control cells were treated with BSA or 0.2 mM PA for 12 h. ER Mg^2+^ signal was visualized by co-staining the cells using ER Tracker and Mag-Fluo4-AM. Shown were representative fluorescent images, scale bar, 10 μm. **b** Mg^2+^ signal in mitochondria. Cells were treated as in **a**. Mitochondrial Mg^2+^ signal was visualized by co-staining the cells using MitoTracker and Mag-Green-AM. Shown were representative fluorescent images, scale bar, 10 μm. **c** Oxygen consumption rate (OCR) measurement. Cells were treated as in **a**. Oligomycin (Oligo, 2 μM), FCCP (1 μM) and rotenone/antimycin (ROT/AA, 0.5 μM) were added as indicated. **d** AIF staining. MLKL-KO Hepa 1–6 and control cells were pretreated with Mg^2+^ (MgCl_2_, 10 mM) or *N*-acetyl-L-cysteine (NAC, 2 mM) for 1 h followed by treatment with BSA or 0.2 mM PA for 12 h. Left, representative images, scale bar, 10 μm. Arrows indicate AIF staining in the nucleus. Right, quantification of nuclear AIF positive cells. Data are represented as means ± SEM. Two-tailed Student’s *t*-test was used for statistical analysis. ns, not significant; *P < 0.05, **P < 0.01, ***P < 0.001.

It remains unclear how MLKL deficiency impairs ER function in HCC cells. A previous study demonstrated that activated MLKL can be translocated to the ER membrane to directly initiate the activation of ER stress signaling in a RIPK1/3-MLKL axis-dependent manner^21^. We were intrigued to understand the subcellular localization of MLKL in a RIPK3-deficient context. Consistent with the previous notion, MLKL expression was detected in the ER membrane fraction (Supplementary Fig. S5a). MLKL on the membrane could form cation channels preferentially permeable to Mg^2+^ ^23,24^ and hypotonic treatment generates swelling ER^25,26^. This led us to hypothesize that the depletion of MLKL impairs Mg^2+^ hemostasias in the ER and causes ER swelling. To test this hypothesis, a cell permeant Mg^2+^ indicator, Mag-Fluo4-AM, was applied in live cell confocal fluorescence analysis. Indeed, in the aforementioned swelling ER noted in MLKL-deficient cells upon PA treatment, we observed the highly intensified Mg^2+^ signal (Fig. 5a), suggesting the associated Mg^2+^ accumulation and the ER dysfunction.

Mg^2+^ is compartmentalized within the cells and the intracellular Mg^2+^ dynamics between ER and mitochondria regulates mitochondrial bioenergetics^27^. We asked whether the elevated Mg^2+^ accumulation in ER indicates the disrupted Mg^2+^ mobilization. As such, mitochondrial Mg^2+^ was visualized by simultaneous loading of mitochondrial marker MitoTracker Red and the cell permeant Mg^2+^ indicator Mag-Green-AM. Consistent with our hypothesis, MLKL-deficient cells exhibited the decreased mitochondrial Mg^2+^ signaling (Fig. 5b), suggesting the disturbed ER-mitochondrial Mg^2+^ dynamics, namely ER Mg^2+^ release and mitochondrial Mg^2+^ uptake, in MLKL-KO cells. Consistently, seahorse analysis revealed that MLKL-KO cells showed the elevated mitochondrial respiration (Fig. 5c) and mitochondrial ROS production (Supplementary Fig. S5b), suggesting enhanced mitochondrial bioenergetics.

To confirm the causal links between Mg^2+^ dynamics, mitochondrial bioenergetics and PA-induced parthanatos in HCC cells, MLKL-deficient and -proficient Hepa 1–6 cell culture was supplemented with exogenous MgCl_2_ to increase Mg^2+^ entry into mitochondria^27^, which resulted in the reduced mitochondrial ROS (Supplementary Fig. S5b) and decreased nuclear translocation of AIF (Fig. 5d). Moreover, pre-treatment with *N*-acetyl-L-cysteine, a scavenger of ROS, rescued the cells from PA-induced nuclear translocation of AIF (Fig. 5d; Supplementary Fig. S5b).

Collectively, our data suggest a new working model that MLKL is required for maintaining ER-mitochondrial Mg^2+^ dynamics in HCC cells with intrinsic RIPK3 deficiency. MLKL-deficiency in HCC cells increases the cell susceptibility to metabolic stress-induced parthanatos in the liver microenvironment and enhances anti-tumor immune surveillance.

## Discussion

The role of RIPK3-MLKL in necroptosis has been widely investigated in a broad physiological and pathological context, including different types of cancer. Of note, unlike MLKL, RIPK3 is not ubiquitously expressed. Cumulative evidence supports that RIPK3 protein and transcripts are not detectable in some types of primary tumors and cancer cell lines, which has been attributed to the hyper-methylation of the promoter region of *RIPK3*^12,28–30^. A very recent work^12^ and our data (Fig. 1) further show that RIPK3-silenced cell context, such as HCC cells, generates a widely-existing situation with deficiency in necroptotic signaling. These results strongly suggest a dissociation between RIPK3 and MLKL in their roles in cancer and RIPK3-independent roles for MLKL. In fact, the functions of MLKL beyond necroptosis have been increasingly reported, yet most of these studies were carried out in a RIPK3-proficient context^31^, hardly excluding the involvement of necroptosis. For example, MLKL is involved in endosomal trafficking and generation of extracellular vesicles, which appears independent of RIPK3 but this role could be enhanced by RIPK3^32^. In this study, we pay attention to HCCs that are characterized by intrinsic silencing of RIPK3^12^. This context allowed us to reveal a previously unrecognized role of MLKL in circumventing parthanatos to maintain the survival of HCC cells in a high-fat microenvironment in the liver. To the best of our knowledge, it is the first evidence that establishes a link between MLKL and parthanatos. It will be interesting to understand whether this role is limited to a RIPK3-defienct cell context. Our data may provide a possible answer to this question. We have shown that the reconstitution of RIPK3 reverses the level of parthanatos in HCC cells (Supplementary Fig. S3m). Though more evidence is still needed, our results may potentially suggest an interplay between these two modes of cellular demise and RIPK3 being a switch that allows cells to decide which route to take, depending on the specific situation.

Different forms of RCD, despite distinct features, are known for extensive cross-talks, such as shared death initiators, effector molecules, and subcellular sites identified as key mediators in different processes. Of note, the interplay between mitochondria-regulated cell death and energetic metabolism is quite common in cancer. For example, it has been established that mitochondria are central initiators of the intrinsic apoptotic pathway, but they may also participate in other forms of RCD such as necroptosis, ferroptosis^33^ and pyroptosis^34^. In this study, we identify an interplay between ER and mitochondria that is mediated by the Mg^2+^ transportation between the two organelles, which, once disturbed by the metabolic stress in the liver microenvironment, leads to parthanatos. These findings may suggest a new model of the interplay between RCD triggered by ER dysfunction. It should be mentioned that MLKL phosphorylation can also activate ER stress sensors in a RIPK3-dependent manner, during which activated MLKL can be translocated to the ER membrane to directly initiate the activation of ER stress signaling^21^.

Mg^2+^ regulates myriad cellular functions and serves as an intracellular second messenger^35–37^ to regulate cellular metabolism, structure and bioenergetics^38^. It is recently reported that ER-mitochondrial Mg^2+^ dynamics play an important role in metabolic feedback circuits and mitochondrial bioenergetics^27^. However, the molecular mechanism of Mg^2+^ movement across the membrane has not been well elucidated. A previous study has reported that MLKL forms channels permeable to Mg^2+^, Na^+^ and K^+^. Mutant MLKL that ablates the phosphorylation induces weak Mg^2+^ current, while the phosphomimetic mutant MLKL or co-expression of RIPK3 and MLKL significantly enhances the Mg^2+^ current, suggesting that phosphorylation of MLKL by RIPK3 promotes the Mg^2+^ channel function of MLKL, which is interestingly accompanied by the prompted cell death^23^. Very recently, a follow-up study from the same group reports that full-length MLKL exhibits Mg^2+^ channel activity comparable to N-terminal protein that is sufficient to induce oligomerization and trigger cell death^24^. Moreover, alike Mg^2+^, Na^+^ influx occurs at the beginning of MLKL oligomerization, while irreversible plasma membrane damage occurs in the late stage of necroptosis^39^, suggesting MLKL may form selective channels at an early stage of necroptosis and then self-assemble to large pores to cause membrane lysis.

Both necroptosis and parthanatos are regarded as immunogenic cell death highly related with anti-tumor immunity^1^. According to our results, in HCC cells with MLKL high expression, both pathways are defective, which may contribute to the immune evasion of HCC during progression and therapy. Immune checkpoint blockade (ICB) anti-PD-L1 atezolizumab, in combination with the anti-VEGFA bevacizumab, has been approved for the treatment of the advanced-stage HCC^40^, constituting a breakthrough in the treatment of this disease. However, no robust biomarkers predicting response to ICB have been identified in HCC patients. Unlike lung and urothelial carcinomas, a predictive role of PD-L1 expression for ICB treatment in HCC remains elusive^14,41^. The utility of microsatellite instability and tumor mutation burden, two measures associated with neoantigen load, is also limited by the low prevalence in HCC^42–44^. We herein discover that MLKL suppresses anti-tumor immune response in HCC while MLKL depletion enhances the efficacy of anti-PD-1 therapy in the syngeneic HCC models. It will be worthwhile to test whether MLKL may be used as a biomarker predicting response to ICB in a clinical setting.

Together, our findings may make contributions to the advancement of the field in the following aspects. (1) We discover a RIPK3-independent function of MLKL in HCC. (2) We reveal a new working model showing how MLKL modulates ER-mitochondrial Mg^2+^ dynamics to prevent metabolic stress-induced parthanatos and immune surveillance. (3) Our findings provide molecular insights to understand the immune tolerance in HCC and have a potential translational value in patient stratification for the treatment of ICB in HCC.

## Materials and methods

### Clinical specimens and follow-up

The tissue microarray of the Zhongshan cohort, containing samples from 211 patients with HCC who underwent hepatectomy from April 2005 to September 2008 at the Department of Liver Surgery, Zhongshan Hospital, Fudan University (Shanghai, China), was used in the present study. The study obtained ethical approval from the Institutional Review Board of Zhongshan Hospital (B2021–611) and complied with the standards of the Declaration of Helsinki. Informed consent was received from each patient before the research.

All the patients in the Zhongshan cohort of our study received a histopathological confirmation of HCC after hepatectomy. Every patient adopted a standardized follow-up protocol until December 2011. Briefly, patients underwent follow-up visits with computed tomography or abdominal magnetic resonance imaging scans at an interval of 6 months in the first 2 years. Liver function, serum alpha-fetoprotein and abdominal ultrasound were monitored once every 3 months. The endpoint of the study was overall survival (OS). OS was defined as the interval from surgery to death or the last follow-up visit.

### Tissue microarray and immunohistochemistry of patient samples

HCC samples and the corresponding adjacent liver samples used for tissue microarray construction were acquired at the Zhongshan Hospital, Fudan University. Primary antibody was first tittered against normal tissues to identify the dilutions that rendered optimal sensitivity and specificity. Then, each tissue sample was stained with primary antibodies as indicated at 4 °C overnight. Visualization of the results was performed by sequential incubations of target proteins with the components of the Envision-plus detection system (EnVision+/HRP/Mo, Dako) and 3,3′-diaminobenidine. Negative controls were treated in the same manner without incubation of the primary antibodies. Both the scores for the staining intensity of the targeted protein (ranging from 0 to 3) and for the percentage of tumor cell area (ranging from 0 to 100%) were assessed independently by two pathologists who were blinded to the characteristics or prognosis of patients. Discrepancies were resolved by consensus.

### Antibodies and regents

Antibodies against MLKL (D6W1K) (#37705), MLKL (D2I6N) (#14993), Phospho-MLKL(Ser345) (D6E3G) (#37333), Phospho-MLKL(Ser358) (D6H3V) (#91689), RIP3 (E1Z1D) (#13526), XBP1 (E9V3E) (#40435), BIP (C50B12) (#3177), eIF2α (D7D3) (#5324), Phospho-eIF2α(Ser51) (D9G8) (#3398), Poly/Mono-ADP Ribose (E6F6A) (#83732), VDAC (D73D12) (#4866), GAPDH (14C10) (#2118) and β-Actin (13E5) (#4970) were purchased from Cell Signaling Technology. Anti-DFNA5/GSDME (#ab215191) and anti-AIF (#ab32516) were purchased from Abcam. Anti-Calnexin (#10427–1-AP) was purchased from Proteintech. Antibody against Histone H2A Rabbit mAb (#A3692) was purchased from ABClonal. Anti-mouse PD-1 (#BE0146) was purchased from BioXcell.

Sodium palmitate (#S161420) was purchased from Aladin. BSA (Fatty Acid & IgG Free) (#ST025) was purchased from Beyotime. Necrostatin-1 (#S8037), ferrostatin-1 (#S7243), Z-VAD-FMK (#S7023), Birinapant (#S7015), Olaparib (AZD2281) (#S1060), Ceapin-A7 (#E1099), GSK2606414 (#S7307), 4μ8C (HY-19707) (#S7272), and MNNG (#E0157) were obtained from Selleck Chemicals. SYTOX Green and Magnesium Green were purchased from Thermo Fisher Scientific. Recombinant murine and human TNF-α were purchased from Peprotech.

### Plasmids

pSPAX2 (#12260), pMD2.G (#12259) and LentiCRISPRv2 (#52961) plasmids were purchased from Addgene. pcDNA3.1 (#V790–20) was purchased from Invitrogen. pcDNA3.1-hRIPK3-Flag plasmid was a gift from Prof. Sudan He (Suzhou Institute of Systems Medicine, Suzhou, China). pcDNA3.1-MLKL^T357A/S358A^ and pcDNA3.1-MLKL^T357E/S358D^ were gifts from Prof. Zhaobing Gao (Shanghai Institute of Materia Medica, Chinese Academy of Sciences, Shanghai, China).

### Cell lines

HT29, Hepa 1–6, HepG2, Hep3B, SNU449 and SNU475 cells were purchased from the American Type Culture Collection (ATCC). SNU398, SNU423, SNU739, JHH-7 and JHH-2 cells were purchased from Nanjing COBIOER Biotechnology. Huh7 and SK-HEP1 cells were purchased from the Cell Bank of Type Culture Collection of Chinese Academy of Sciences. All cells were cultured and passaged as suppliers suggested. The identity of cell lines was routinely confirmed using short tandem repeat analysis at Genesky Biotechnologies.

For the generation of MLKL-KO cells, sgRNAs targeting MLKL gene were ordered from Sangon and cloned into lentiCRISPRv2 plasmid. The plasmids were transfected into 293T cells along with pSPAX2 and pMD2.G at a ratio of 4:3:1, respectively. Hepa 1–6-luc cells were infected with lentiviral particles containing indicated sgRNAs for 48 h and then selected by puromycin. Single-cell clones were picked and expanded, and the KO efficiency was confirmed by immunoblotting analysis.

Sequences used in this study were as follows:

sgMLKL #1 forward 5'-CACCGGCACACGGTTTCCTAGACGC-3'

reverse 5'-AAACGCGTCTAGGAAACCGTGTGCC-3'

sgMLKL #2 forward 5'-CACCGAACCCCCAGGCCGAAAGTGT-3'

reverse 5'-AAACACACTTTCGGCCTGGGGGTTC-3'

### Cell transfection

For plasmid transfection, cells were plated at 30%–50% confluence and transfected with indicated plasmids using Lipofectamine 2000 Transfection Reagent (#11668019, Invitrogen) according to the manufacturer’s instructions. For siRNA transfection, cells were plated at 30%–50% confluence and transfected with indicated siRNA duplex using lipofectamine RNAiMAX (#13778030, Invitrogen). siRNAs were ordered from Tsingke Biotechnology. The sequences were as follows:

Negative control siRNA (siNC) 5'-AUCUUAUGAAGGACUU-3'

siMLKL #1 5'-CAAACUUCCUGGUAACUCA-3'

siMLKL #2 5'-UCAAGGACGUGAACAGGAA-3'

siMLKL #3 5'-GCAAUAGAUCCAAUAUCUG-3'

### Animal models

C57BL/6 mice and BALB/c nude mice of 6–8 weeks old (female) were obtained from Shanghai Lingchang Biotechnology. To generate heterotopic tumor models, 1 × 10^6^ Hepa 1–6-luc cells or derived sublines suspended in 100 μL of DMEM medium were injected subcutaneously to the right flank of 6–8-week-old athymic nude or C57 BL/6 mice. To generate orthotopic tumor models, 2 × 10^6^ Hepa 1–6-luc cells or derived subline suspended in 20 μL of DMEM medium were surgically implanted into the livers in athymic nude or C57BL/6 mice. Tumor-carrying mice were randomly assigned to experimental groups. Animal studies were performed under the approval of the Institutional Animal Care and Use Committee at Shanghai Institute of Materia Medica, Chinese Academy of Sciences.

For in vivo imaging, mice were anesthetized using Zoletil (intraperitoneally). After 5 min mice were moved into an IVIS Lumina II instrument (Caliper Life Sciences) and luminescence was measured using 1 min exposure.

### Analysis of tumor-infiltrating lymphocytes

Tumors were dissected, digested with digest solution containing Dnase I and collagenase V, and passed through 70-μm nylon strainers (BD Biosciences) to obtain single-cell suspensions. Cells were stained with anti-CD16/CD32 (500× dilution) (BD Biosciences) to block Fc receptors followed by staining with Fixable Viability Dye efluor 780 (1000× dilution) (eBioscience). Surface antigens or intracellular proteins were stained followed by manufacturer’s instructions. Cell analysis was performed on Fortessa (Becton Dickinson), and data were analyzed using the FlowJo software.

### Immunohistochemistry analysis of mouse tumors

The tumor tissues were fixed with 4% paraformaldehyde immediately after mice were sacrificed. Subsequent immunohistochemistry analysis was performed by Shanghai Zuocheng Biological Technology. Images of sections were captured with NanoZoomer S210 (Hamamatsu) and further analyzed using NDP.View 2 software. The CD8^+^ cell density was analyzed by ImageJ.

### Immunoblotting analysis

Protein lysis was separated using SDS-PAGE, transferred into a nitrocellulose membrane (Immobilon-P, Millipore), and then blocked for 1 h with 5% BSA in 1× Tris-buffered saline with Tween-20 (TBST) (25 mM Tris, 150 mM NaCl, 2 mM KCl, pH 7.4, supplemented with 0.1% Tween-20). Membranes were immunoblotted with primary antibodies overnight at 4 °C. After washing with TBST, membranes were blotted with horseradish peroxidase-conjugated anti-rabbit IgG (1:2000 dilution) or anti-mouse IgG (1:2000 dilution) antibodies for 1 h at room temperature. Blots were visualized by Pro e-BLOT Touch Imager according to the manufacturer’s instructions.

### Analysis of cell death

For cell death analysis, cells were treated with BSA or PA for 24 h. For SYTOX Green uptake assay, cells were harvested after trypsin digestion, washed with HBSS (Thermo Fisher Scientific) twice and then incubated with SYTOX Green nucleic acid stain (#S7020, Thermo Fisher Scientific) for 15 min. Cell death was measured based on SYTOX Green fluorescence (excitation 485 nm, emission 520 nm). For LDH leakage analysis, culture media was collected and LDH release was examined using the LDH-Glo Cytotoxicity assay (Promega) according to the manufacturer’s instruction.

### Immune cell activation assay

Cells treated with BSA or 0.2 mM PA for 24 h and the supernatant was collected as the conditioned medium. For BMDC activation assay, BMDCs were treated with the conditioned medium at 1:100 dilution and then collected into test tubes. Cells were stained with anti-CD16/CD32 (500× dilution) (BD Biosciences) to block Fc receptors followed by staining with Fixable Viability Dye eFluor 780 (1000× dilution) (eBioscience), CD11b (#101206, Biolegend), CD11c (#117329, Biolegend), CD80 (#553769, BD Biosciences) and CD86 (#105014, Biolegend) antibodies. For CD8^+^ T cell activation assay, CD8^+^ T cells were isolated from the spleen of mouse using EasySep Mouse CD8^+^ T Cell Isolation Kit (#5371.2, StemCell) and treated with IL-2 (#212–12–20, PeproTech), IL-7 (#217–17, PeproTech), IL-15 (#210–15, PeproTech), anti-CD3 (#300465, Biolegend), anti-CD28 (#102116, Biolegend) and the conditioned medium at 1:100 dilution for 72 h. Cells were then stained with anti-CD16/CD32 (500× dilution) (BD Biosciences) to block Fc receptors followed by staining with Fixable Viability Dye eFluor 780 (1000× dilution) (eBioscience), CD44 (#740215, Biolegend), GranzymeB (#372208, Biolegend) and IFNγ (#17–7311–82, Invitrogen) antibodies.

### Transmission electron microscopy

Cells were scraped from 100-mm culture dish and transferred into centrifuge tubes. Then the cells were centrifuged at 1000 rpm for 5 min and washed with PBS twice. Pellets were fixed in 2.5% electron microscopy specific glutaraldehyde at 4 °C overnight. Transmission electron microscopy was conducted by Biomisp (Wuhan, China).

### Confocal microscopy

For mitochondrial ROS staining, live cells were first stained with 1 μM MitoSox Red (#M36008, Invitrogen) at 37 °C for 10 min and then fixed with 4% paraformaldehyde for 15 min. For AIF staining, cells were fixed with 4% paraformaldehyde for 15 min, permeabilized with 0.5% Triton X-100 for 15 min, and then treated with AIF antibody (#ab32516, Abcam). For Mg^2+^ staining, cells seeded in a cover-glass slide chamber were stained with Mag-Fluo4-AM (#M14206, Invitrogen) or Mag-Green-AM (#M3735, Invitrogen). Images were captured using a laser scanning confocal microscope (Fluoview FV 1000) with a UPlansApo 100×/1.40 oil objective (Olympus) at room temperature and processed using ImageJ.

### ER isolation

ER was isolated using ER isolation kit (ER0100–1KT, Sigma Aldrich) according to the manufacturer’s instructions.

### RNA-seq analysis

RNA-seq analysis was conducted by Shanghai Majorbio Bio-pharm Technology. In brief, total RNAs were extracted and RNA-seq libraries were prepared using Illumina RNA-Seq Preparation Kit and sequenced by an Ilumina Hiseq sequencer. RNA-seq reads were mapped using Hisat2 with default settings. Transcript assembly and transcript abundance quantification were carried out using feature Counts. Differential expression analysis was performed using DESeq2 R package. KEGG and GO pathway enrichment analysis was performed using ClusterProfiler R package.

### Statistical analysis

Data were expressed as means ± SEM. Statistical significance was determined using one-way ANOVA with Dunnett multiple-comparisons test post hoc or Student’s *t*-test as appropriate. Log-rank test was conducted for survival analysis. Differences were considered to be statistically significant at *P* < 0.05. Statistical calculations were performed with GraphPad Prism.

## Supplementary information

Supplementary Information
