# Supplementary material for: A RIPK3-independent role of MLKL in suppressing parthanatos promotes immune evasion in hepatocellular carcinoma
Source: Cell Discov. 2023 Jan 17;9:7. doi: 10.1038/s41421-022-00504-0 (PMC9845215; doi:10.1038/s41421-022-00504-0)
Supplement: Supplementary file 1 — Supplementary Information [file 41421_2022_504_MOESM1_ESM.pdf]

Supplementary Information

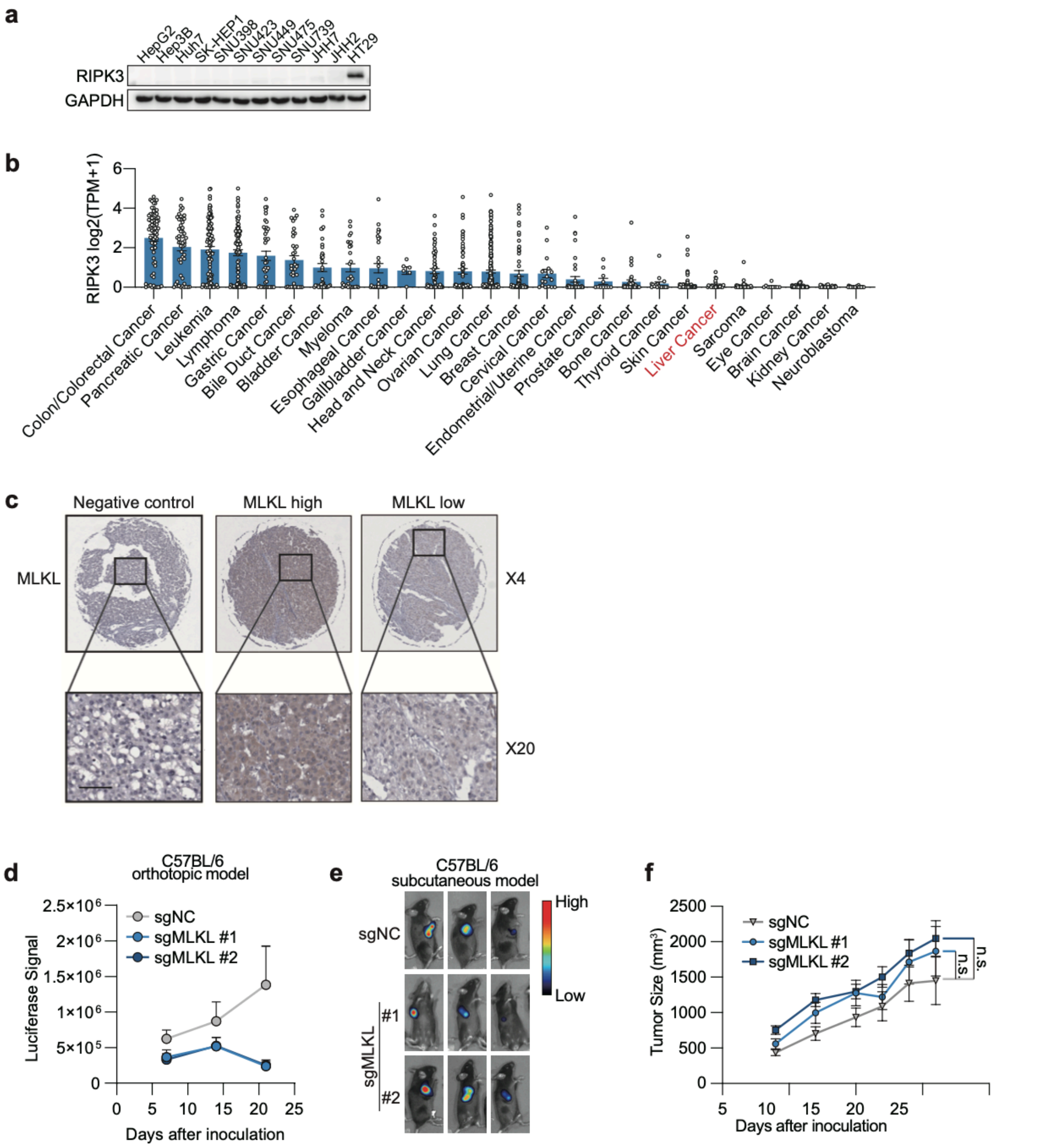

Supplementary Fig. S1 MLKL deficiency impairs orthotopic growth of HCC tumors. (a) RIPK3 protein level in a panel of HCC cell lines. Colon cancer cell line HT29 was used as a positive control. **(b)** RIPK3 expression in different types of cancer cells. Data were from CCLE. **(c)** Typical pictures of stained MLKL signal in HCC tissues. MLKL was stained using immunohistochemical analysis. Scale bar, 50 μm. **(d)** Orthotopic tumor growth of MLKL-knockout and control Hepa1-6-luc cells described in Fig. 1g. Shown were quantification of luminescent signals. **(e)** Subcutaneous tumor growth of MLKL-knockout and control Hepa 1-6-luc tumors. Tumor growth was monitored by whole animal imaging. Shown were representative luminescent images. **(f)** Subcutaneous tumor growth curves of MLKL-knockout and control Hepa

1-6 tumors. Tumor growth was monitored by measuring tumor volume (n = 10). Data are represented as mean ± SEM. Two-tailed Student's t test was used for statistical analysis. ns, not significant; $^{*}p < 0.05$, $^{**}p < 0.01$, $^{***}p < 0.001$.

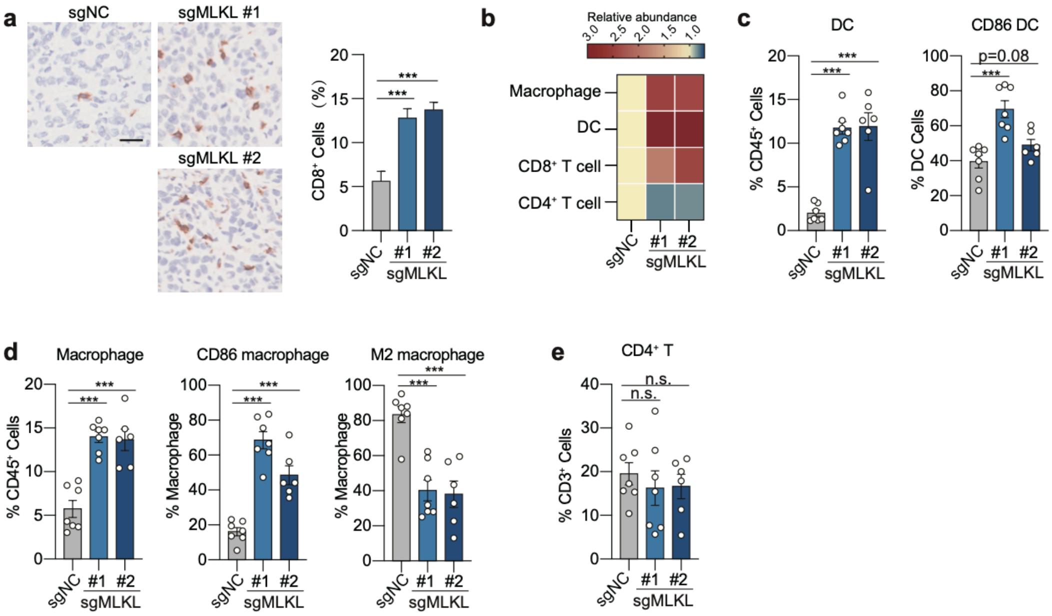

Supplementary Fig. S2 MLKL deficiency activates anti-tumor immunity in HCC tumors. (a) CD8 staining using immunohistochemistry analysis. MLKL-knockout Hepa1-6-luc and control cells were orthotopically implanted in C57BL/6 mice. Tumor tissues were collected for analysis. Left, representative images, scale bar, 25 μm. Right, quantification of $CD8^+$ cells. **(b)** Heatmap of relative abundance of macrophage, DC, $CD8^+$ T and $CD4^+$ T cells. MLKL-knockout Hepa1-6 and control cells were orthotopically implanted in C57BL/6 mice and tumor infiltrating immune cells were assessed by flow cytometry analysis. The percentage of immune cells were normalized based on control tumors. **(c)** Percentage of DC cells in immune cells (left) and $CD86^+$ DC cells in DC cells (right). Tumor infiltrating immune cells were analyzed as (b). **(d)** Percentage of macrophage cells in immune cells (left), $CD86^+$ M1 macrophage cells in macrophage cells (middle) and M2 macrophage cells in macrophage cells (right). Tumor infiltrating immune cells were analyzed as (b). **(e)** Percentage of $CD4^+$ T cells in T cells. Tumor infiltrating immune cells were analyzed as (b). Data are represented as mean ± SEM. Two-tailed Student's t test was used for statistical analysis. ns, not significant; $^{*}p < 0.05$, $^{**}p < 0.01$, $^{***}p < 0.001$.

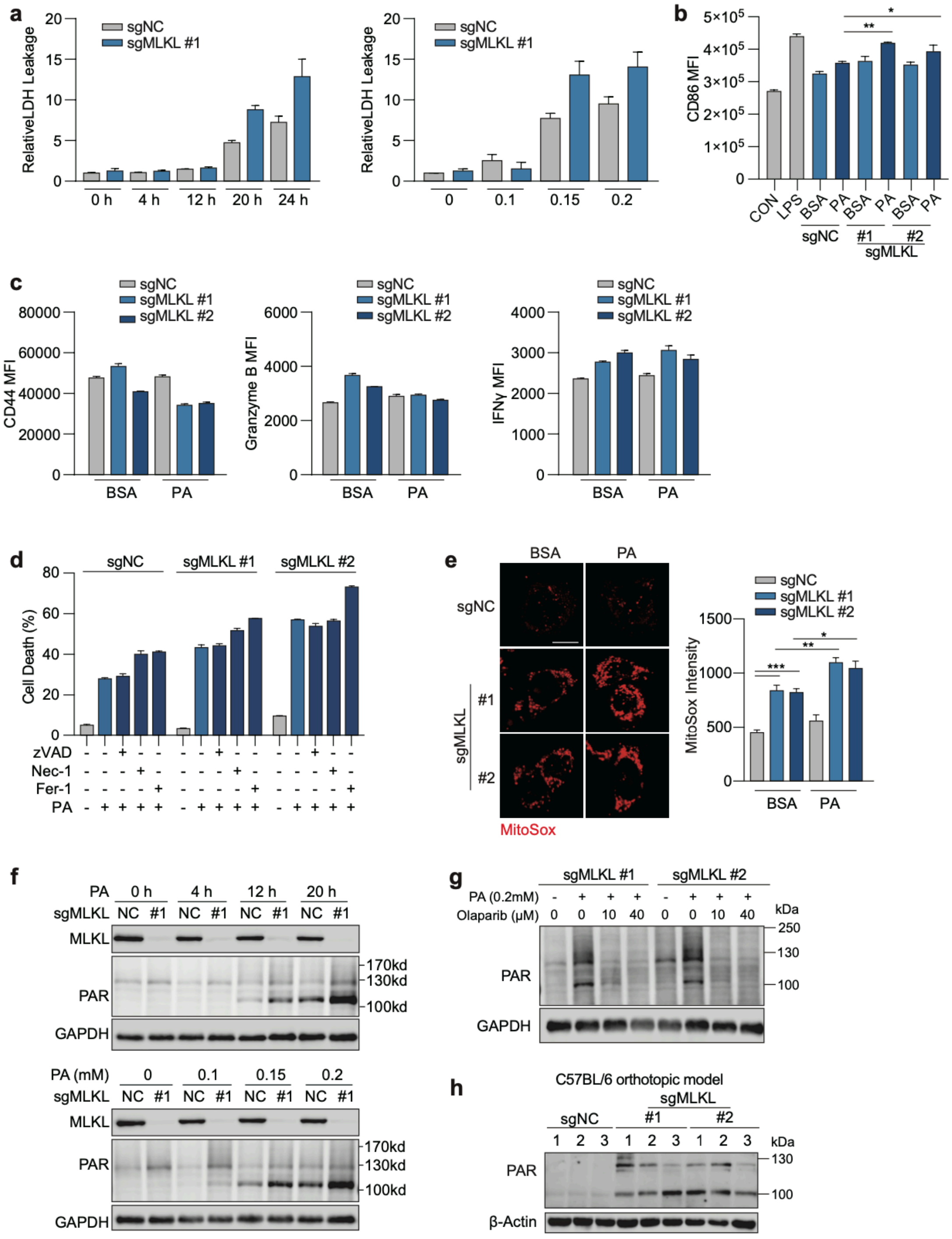
a
sgNC
sgMLKL #1
RelativeLDH Leakage
0 h
4 h
12 h
20 h
24 h
0
0.1
0.15
0.2
b
CD86 MFI
CON
LPS
BSA
PA
sgNC
#1
#2
sgMLKL
c
sgMLKL #2
CD44 MFI
Granzyme B MFI
IFNγ MFI
d
Cell Death (%)
zVAD
Nec-1
Fer-1
e
MitoSox
MitoSox Intensity
f
MLKL
PAR
GAPDH
170kd
130kd
100kd
PA (mM)
g
PA (0.2mM)
Olaparib (μM)
kDa
h
C57BL/6 orthotopic model
β-Actin

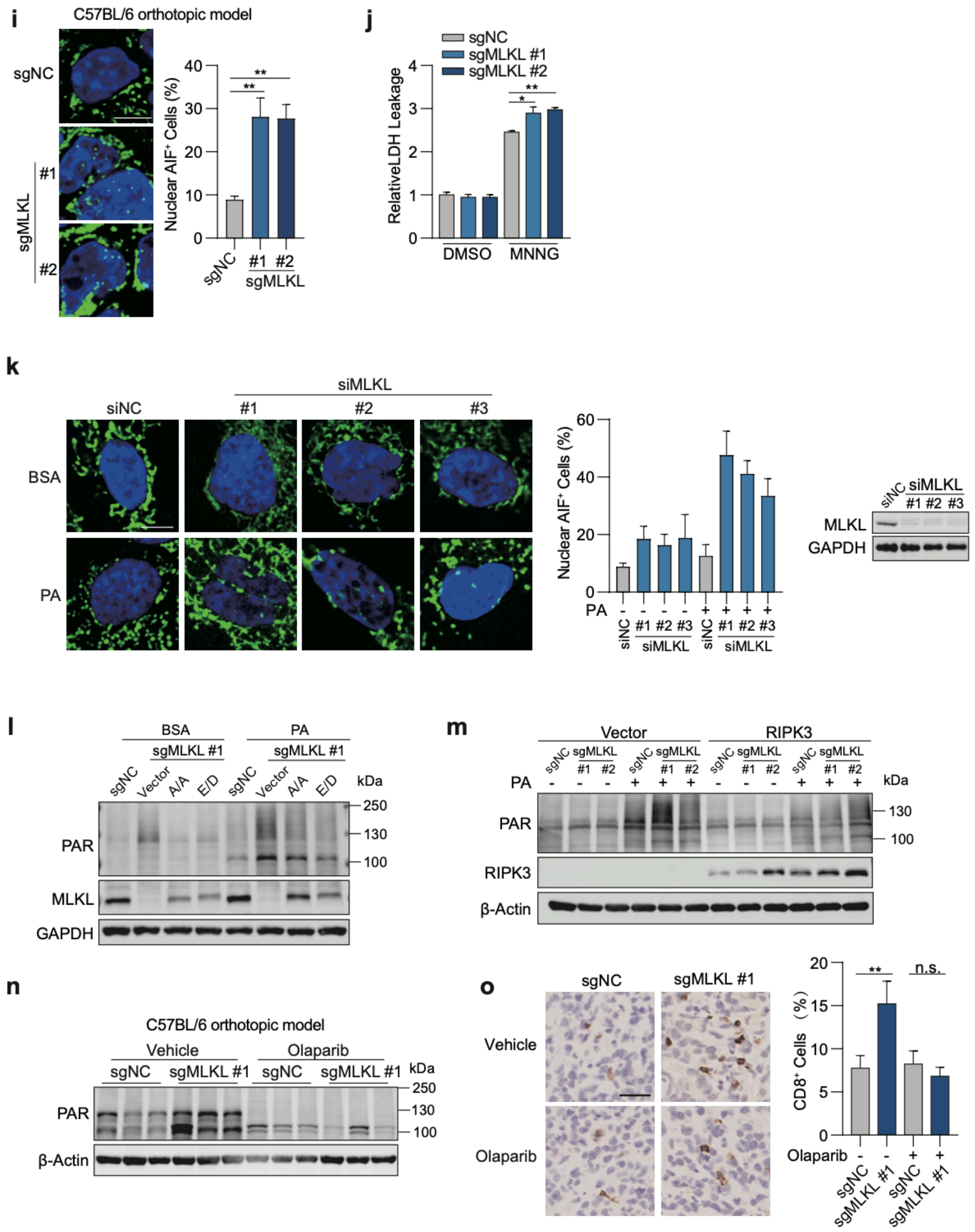

Supplementary Fig. S3 MLKL deficiency enhances metabolic stress-induced parthanatos. (a) Cell death induced by PA. MLKL-knockout and control Hepa1-6 cells were treated with 0.2 mM PA for indicated time (left) or at indicated concentration for 24 h (right). Cell death was measured by LDH leakage. **(b)** Dendritic cells activation described in Figure 3d. CD86 expression of BMDCs was analyzed using flow cytometry analysis. **(c)** Co-culture assay of $CD8^+$ T cells activation. MLKL-knockout and control cells were treated with or without PA (0.2 mM, 24 h) and supernatant was collected as conditioned medium. $CD8^+$ T cells isolated from the spleen of C57BL/6 mice were treated with conditioned medium for 72 h. CD44, Granzyme B and IFNγ expression was analyzed

through flow cytometry analysis. **(d)** Cell death analysis. MLKL-knockout and control Hepa1-6 cells were pre-treated with apoptosis inhibitor z-VAD-FMK (50 μM), necroptosis inhibitor Nec-1 (20 μM) or ferroptosis inhibitor Fer-1 (10 μM) respectively for 2 h, and then exposed to PA for 24 h. **(e)** Mitochondrial ROS production. MLKL-knockout and control Hepa1-6 cells were treated with or without PA for 12 h. Mitochondrial ROS was monitored by MitoSox staining. Left, representative images, scale bar, 10 μm. Right, quantification of fluorescence intensity. **(f)** Accumulation of PAR polymer. MLKL-knockout and control Hepa1-6 cells were treated with 0.2 mM PA for indicated time (left) or at indicated concentration for 20 h (right) and then analyzed by immunoblotting. **(g)** Accumulation of PAR polymer. MLKL-knockout Hepa1-6 cells were treated with 0.2 mM PA and/or Olaparib at indicated concentrations for 20 h. **(h)** PAR polymer accumulation of orthotopic tumors. MLKL-knockout and control Hepa1-6 orthotopic tumors described in Fig. 1g were subjected to immunoblotting analysis. **(i)** AIF staining of tumors in (h). AIF was stained using immunofluorescence. Left, representative images, scale bar, 10 μm. Right, quantification of nuclear AIF positive (AIF+) cells. **(j)** Cell death induced by MNNG. MLKL-knockout and control Hepa 1-6 cells were treated with MNNG (1 mM, 24 h). Cell death was measured by LDH leakage. **(k)** AIF staining. SK-HEP1 cells were transfected with indicated siRNAs for 48 h and then exposed to PA (0.4 mM, 20 h). AIF was stained using immunofluorescence. Left, representative images, scale bar, 10 μm. Middle, quantification of nuclear AIF positive (AIF^+) cells. Right, MLKL knockdown efficiency assessed by immunoblotting analysis. **(l)** Accumulation of PAR polymer. MLKL-knockout Hepa1-6 cells were transfected with empty vector, pcDNA3.1-$MLKL^{A/A}$ or pcDNA3.1-$MLKL^{E/D}$ for 24 h followed by PA treatment (0.2 mM, 20 h) and then analyzed by immunoblotting. **(m)** Accumulation of PAR polymer. MLKL-knockout and control Hepa1-6 cells were transfected with empty vector or pcDNA3.1-hRIPK3-Flag for 24 h followed by PA treatment (0.2 mM, 20 h) and then analyzed by immunoblotting. **(n)** PAR polymer accumulation in orthotopic tumors. MLKL-knockout and control Hepa1-6 orthotopic tumors described in Fig. 3h were subjected to immunoblotting analysis. **(o)** CD8 staining in orthotopic tumors. MLKL-knockout and control Hepa1-6 orthotopic tumors described in Fig. 3h were subjected to immunohistochemistry analysis of CD8. Left, representative images, scale bar, 25 μm. Right, quantification of $CD8^+$ T cells. Data are represented as mean ± SEM. Statistical analyses were performed using two-tailed Student's t

test. ns, not significant. $^{*}p < 0.05$, $^{**}p < 0.01$, $^{***}p < 0.001$.

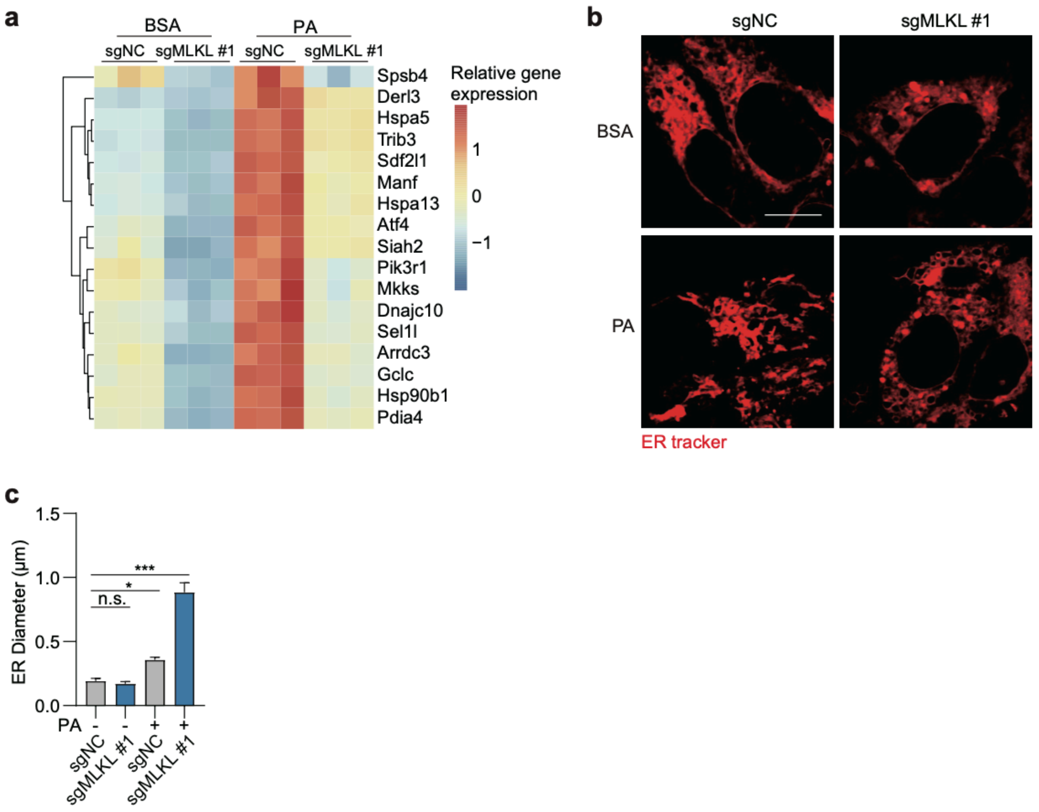

Supplementary Fig. S4 MLKL deficiency leads to ER dysfunction in HCC cells. (a) Heatmap of mRNA expression of ER relative genes in cells described in Fig. 4a. **(b)** Morphological changes of ER. MLKL-knockout and control Hepa1-6 cells were treated with or without PA (0.2 mM, 12 h). ER were stained with ER tracker. Shown were representative fluorescent images, scale bar, 10 μm. **(c)** Quantification of ER diameter described in Fig. 4d. All data represent means of indicated technical replicates; error bar represent SEM. Statistical analyses were performed using two-tailed Student's t test. ns, not significant. $^{*}p < 0.05$, $^{***}p < 0.001$.

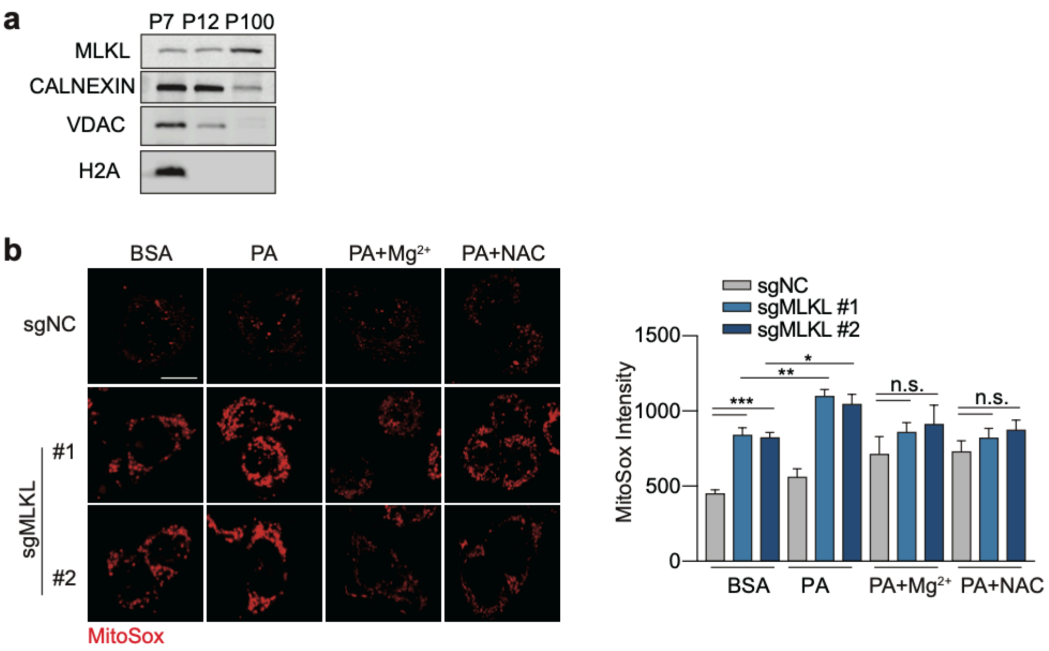

Supplementary Fig. S5 MLKL deficiency disrupts Mg^{2+} homeostasis in the ER. (a) Subcellular localization of MLKL in HCC cells. ER was isolated from Hepa 1-6 cells and sequential fractions were analyzed by immunoblotting. Calnexin, an ER maker; VDAC, a mitochondrial marker. H2A, a nuclear marker. **(b)** Mitochondrial ROS production. MLKL-knockout and control Hepa1-6 cells were pretreated with Mg^{2+} ($MgCl_2$, 10 mM) or NAC (2 mM) for 1 h before challenged with 0.2 mM PA for 12 h. Mitochondrial ROS was monitored by MitoSox staining. Left, representative images, scale bar, 10 µm. Right, quantification of fluorescence intensity. All data represent means of indicated technical replicates; error bar represent SEM. Statistical analyses were performed using two-tailed Student's t test. ns, not significant. $^{*}p < 0.05$, $^{**}p < 0.01$, $^{***}p < 0.001$.
